# Adaptive Data Selection-Based Machine Learning Algorithm for Prediction of Component Obsolescence

**DOI:** 10.3390/s22207982

**Published:** 2022-10-19

**Authors:** Kyoung-Sook Moon, Hee Won Lee, Hongjoong Kim

**Affiliations:** 1 Department of Mathematical Finance, Gachon University, 1342 Seongnamdaero, Sujeong-gu, Seongnam-si 13120, Gyeonggi-do, Korea; 2 Department of Mathematics, Korea University, 145 Anam-ro, Seongbuk-gu, Seoul 02841, Korea

**Keywords:** component obsolescence, diminishing manufacturing sources and material shortages, forecasting, machine learning, unsupervised clustering

## Abstract

Product obsolescence occurs in the manufacturing industry as new products with better performance or improved cost-effectiveness are developed. A proactive strategy for predicting component obsolescence can reduce manufacturing losses and lead to customer satisfaction. In this study, we propose a machine learning algorithm for a proactive strategy based on an adaptive data selection method to forecast the obsolescence of electronic diodes. Typical machine learning algorithms construct a single model for a dataset. By contrast, the proposed algorithm first determines a mathematical cover of the dataset via unsupervised clustering and subsequently constructs multiple models, each of which is trained with the data in one cover. For each data point in the test dataset, an optimal model is selected for regression. Results of empirical experiments show that the proposed method improves the obsolescence prediction accuracy and accelerates the training procedure. A novelty of this study is that it demonstrates the effectiveness of unsupervised clustering methods for improving supervised regression algorithms.

## 1. Introduction

The acceleration of technological development is rendering current technological innovation obsolete. Particularly, innovations in electronic devices based on semiconductors have exacerbated the problem of component obsolescence. However, accurate prediction of obsolescence can significantly reduce costs associated with product design and production. Therefore, preventive and preemptive technology development and management based on the prediction of future obsolescence levels is necessary. Many studies have focused on the concept and management of obsolescence, and the obsolescence problem is referred to as the diminishing manufacturing sources and material shortages (DMSMS) problem (see [1,2]).

Jennings et al. [3] reported that as obsolescence increases, a proactive approach offers more advantages than a reactive approach, which requires more time and materials than the former to manage obsolescence. The key to proactive management is the accurate prediction of the risk of component obsolescence. As outlined in Section 2.1, numerous studies have focused on predicting obsolescence based on mathematical models, and recently, methods using machine learning (ML) have been proposed, as summarized in Table 1.

In this study, we aim to predict the obsolescence date of electronic parts based on the data. In order to accurately predict the obsolescence of parts, we propose an adaptive data selection (ADS)-based ML method. The newly presented ADS method first divides the input dataset into subsets of similar data. Subsequently, when a new data point is inserted, it selects one optimal subset and performs learning and prediction with it. Existing clustering methods, such as the *k*-means method, do not allow common data between groups, whereas the ADS method classifies data of the same nature into one group while allowing common data between the groups. As a case study, we apply several ML methods, introduced in Section 2.2, with ADS to predict the obsolescence date of electronic diodes. As discussed in Section 5, the prediction results of learning using ADS are considerably more accurate than those of the method that does not use clustering or the *k*-means method.

The main improvements of this paper are as follows. Firstly, the obsolescence date is accurately predicted based on the discontinued electronic component data using ML methods. Secondly, the accuracy of prediction is improved by dividing the dataset into subsets according to the characteristics, finding parameters suitable for each subset and applying a learning algorithm. Thirdly, in order to offset the shortage of data on discontinued electronic components and to increase the accuracy near the boundary of subsets, a new ADS method is proposed to improve accuracy and efficiency.

The remainder of this paper is organized as follows. Section 2.1 and Section 2.2 present the previous studies on obsolescence prediction problems and the ML algorithms used in the numerical experiments, respectively. The proposed ADS-based method is described in Section 3. The statistics of the data and the hyperparameters are presented in Section 4. The accuracy measures and experimental results are presented and discussed in Section 5. The conclusions are discussed in Section 6.

## 2. Background

### 2.1. Literature Review

According to Trabelsi et al. [2], the causes of component obsolescence can be classified by technological development, availability, material shortage, economic feasibility, and environmental factors. It is more economical and efficient to detect and manage component obsolescence problems caused by these various causes in advance. Unlike reactive management, proactive management identifies and prioritizes components at risk of obsolescence. For proactive management, in order to suggest resolutions before components are discontinued, it is necessary to accurately predict the risk of component obsolescence. Many studies on predicting the risk of component obsolescence have been conducted, and they can be divided into two categories, one using a mathematical model based on statistical techniques and the other using ML, as summarized in Table 1.

Mathematical models based on various statistical methods have been studied. Solomon et al. [4] presented a life-cycle forecasting model based on the Gaussian distribution methodology for forecasting the years to obsolescence for electronic parts of integrated circuits. Sandborn et al. [5] proposed combining life-cycle curve forecasting and data mining of historical last-order or last-ship dates. Rojo et al. [6] reviewed the literature on the problem of obsolescence published before 2009. Sandborn et al. [7] proposed a method based on data mining of historical records to forecast procurement lifetime and obsolescence date, and Ma and Kim [8] predicted life cycle curves based on a time series model without evolutionary parametric drivers. Trabelsi et al. [2] developed a method based on probability distribution to model the degree of obsolescence as a function of time. Mastrangelo et al. [9] proposed the conditional Weibull distribution method to determine the likelihood of a component becoming obsolete.

Recently, ML and deep learning (DL) algorithms, which can learn from vast amounts of data, have yielded promising results in various fields [10,11]. However, to the best of the authors’ knowledge, studies using ML to predict the obsolescence of parts remain limited. Among them, Jennings et al. [3] tested the effectiveness of random forest (RF), neural network, and support vector machine algorithms for obsolescence risk and life-cycle forecasting. Grichi et al. [12] used the RF method for obsolescence forecasting and improved it by combining it with a genetic algorithm [13]. The limited success of ML or DL methods for obsolescence prediction can be attributed to the scarcity of available data. To address this problem, Moon et al. [14] proposed using the *k*-means clustering method before applying a ML algorithm and employed this approach to improve the forecasting accuracy of the obsolescence date of electronic diodes.

**Table 1:** A summary of recent studies on predicting the risk of component obsolescence. (RF: random forest, NN: neural network, SVM: support vector machine, LR: logistic regression, GB: gradient boosting, GA: genetic algorithm, DT: decision tree, DNN: deep neural network, RNN: recurrent neural network).

Category	Authors (Year)	Application (Target)	Method
Mathematical	Solomon et al. (2000) [4]	Integrated circuits (years to obsolescence)	Gaussian distribution
models	Sandborn et al. (2007) [5]	Flash memory/DRAM (life cycle curve)	Curve fitting
	Sandborn et al. (2011) [7]	Electronic parts (obsolescence dates)	Linear regression
	Ma and Kim (2017) [8]	Flash memory (life cycle curve)	Time series model
	Trabelsi et al. (2021) [2]	Smartphones (obsolescence probability density)	Statistical test
	Mastrangelo et al. (2021) [9]	Electronic parts (obsolescence probability)	conditional probability method
Machine	Jennings et al. (2016) [3]	Cellphones (obsolescence dates)	RF/NN/SVM
learning	Grichi et al. (2017) [12]	Cellphones (obsolescence dates)	RF/Multiple linear regression
	Grichi et al. (2018) [13]	Cellphones (obsolescence risk)	LR/GB/RF with GA
	Moon et al. (2022) [14]	Electronic diodes (obsolescence dates)	DT/RF/GB/DNN/RNN with *k*-means clustering

### 2.2. Learning Models

This section briefly reviews the ML and DL methods used in the numerical experiments. ML extracts desired information from data based on statistics and artificial intelligence. It was first proposed in the 1950s, and it has been actively studied since 2000 and used in a variety of fields, including computer science and finance. ML can be classified into supervised and unsupervised learning, and the former can be categorized into classification and regression [15,16].

In this study, we use a regression model to predict future obsolescence dates. Representative regression models include the *k*-nearest neighbors, linear regression, support vector machines, and decision trees (DTs). We use DT in Section 2.2.1 and two ensemble models with a DT as the basic element, namely the RF method in Section 2.2.2 and the gradient boosting (GB) method in Section 2.2.3. Additionally, the results are compared with those obtained using a deep neural network (DNN) and recurrent neural network (RNN) in Section 2.2.4, which are DL models with multilayered neural networks.

#### 2.2.1. Decision Tree

The DT method is a method of learning that involves repeated creation of a binary tree or hierarchical division of a domain. Prediction on new data involves determining the location of the given data among the divided features, finding leaf nodes, and using the average value of the training data of the found leaf nodes to make a prediction. Learning proceeds in the direction that maximizes the information gain in each branch, which can be expressed as follows:

(1) $$Info=E\left(parent\right)-\sum_{i=1}^{n}\frac{N_{i}}{N_{p}}E\left(children_{i}\right),$$

where *parent* and *children* denote before and after branch, respectively; and $N_{i}$, $N_{p}$, and *n* represent the number of samples of the *i*-th child node, number of samples of the *p*-th parent node, and number of children, respectively. Here, *E* is the entropy, which is defined as

(2) $$E=-\sum_{i}p_{i}\log_{2}\left(p_{i}\right),$$

where $p_{i}$ is the proportion of data belonging to category *i* among the data in an area.

In this process, if a leaf node proceeds until it becomes a pure node comprising a single target, the model may become complex and result in overfitting. Therefore, the *max_depth* and *max_leaf_nodes* variables are used to limit the maximum depth of the tree and the maximum number of leaves, respectively. We mitigate the overfitting of the training data by pre-pruning using the *min_samples_split* and *min_samples_leaf* variables (Table 6).

DTs are easy to understand by model visualization and do not require preprocessing steps such as the regularization of the data size and characteristics. However, even with pre-pruning, the training data can be overfitted, and thus, the ensemble methods introduced in the following sections are preferred.

#### 2.2.2. Random Forest

To compensate for the overfitting problem associated with DT, an RF randomly creates many DTs and averages the results. First, we determine the number of trees, randomly create bootstrap samples, and create a DT for each generated dataset. Here, not all of the features are considered; instead, the best test among randomly selected features is determined. The number of DTs and number of features are modified using the *n_estimator* and *max_features* variables, respectively, as listed in Table 6.

In previous studies such as [3,12,13], the RF method is the most extensively used ML method. It addresses the limitation of DT and offers the advantages of high performance and non-requirement of scaling data. Although averaging more trees can reduce overfitting and create a stabler model, this requires more memory and computation time.

#### 2.2.3. Gradient Boosting

GB is an ensemble method that creates an improved model by combining multiple DTs. However, unlike the RF method, it creates a tree to compensate for the error of the previous tree. GB requires less memory and prediction time than the RF method; however, it is more sensitive to parameter values than the latter. In addition to the increase in *n_estimator*, which denotes the number of trees, that in *learning_rate*, which controls the degree of error correction of the previous tree, increases the model complexity.

#### 2.2.4. Deep Learning

DL is a method inspired by the structure and function of the human brain, which essentially repeats tasks while making minor adjustments based on learning to improve results. It is a class of ML methods. DNN expresses various concepts in a hierarchical structure and offers excellent capabilities and flexibility. However, there are still limitations arising from the repeatability of DL algorithms, complexity of increasing the number of layers, and the large amount of data required for network training. Additionally, compared to conventional ML, a DL algorithm requires more data to understand a certain problem, and if the amount of data is insufficient, it performs poorly.

**Figure 1:**
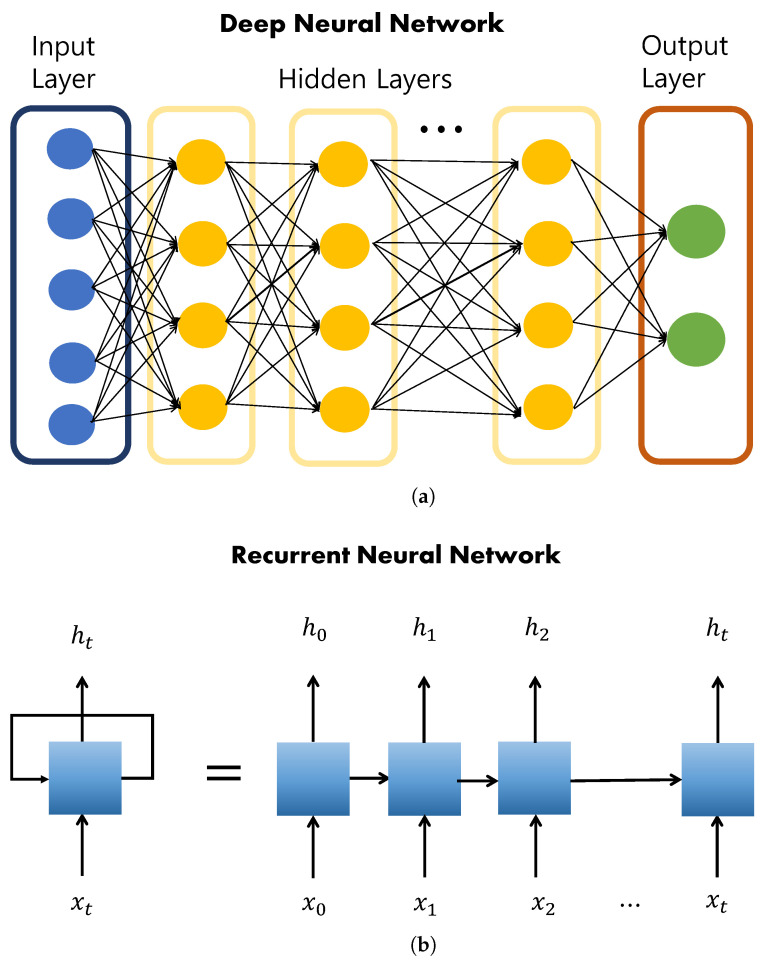
Schematic of (**a**) DNN and (**b**) RNN.

The most basic element in DL is a neural network, which represents the neuronal connection structure among the biological characteristics of the human brain. A network structure modeled after this neural network is called an artificial neural network. An artificial neural network receives learning data from the input layer, processes them using several hidden layers, and outputs the final result through the output layer. A structure in which these neural networks are superimposed is called a DNN, as shown in Figure 1a. A structure that uses an internal cyclic structure to reflect past learning in current learning via weights is called an RNN, as shown in Figure 1b.

## 3. Proposed Method

Given a dataset, an ML algorithm identifies its parameters and hyperparameters and builds one model with them. However, some data show various characteristics or patterns in it, and one set of parameters and hyperparameters may not be sufficient for proper representation of the data, which leads to inaccurate prediction. The current study thus proposes to split the dataset into several subsets and to identify parameters and hyperparameters for each subset. Then one can construct a *group* of ML models, each of which best fits one of the subsets. We introduce two unsupervised clustering methods in Section 3.1 to split the dataset to its subsets. One is *k*-means clustering in Section 3.1.1, whose subsets are mutually disjoint, and the other is ADS in Section 3.1.2, whose subsets have non-empty intersections. We subsequently propose an approach to combine the regression-based supervised learning methods presented in Section 2.2 with unsupervised clustering methods in Section 3.2. For each data point in the test dataset, one identifies a subset that best describes its characteristic or pattern and applies the ML model trained with that subset for the prediction. This strategy is shown to improve the prediction accuracy in Section 5.

### 3.1. Unsupervised Clustering

#### 3.1.1. *k*-Means Clustering

**Figure 2:**
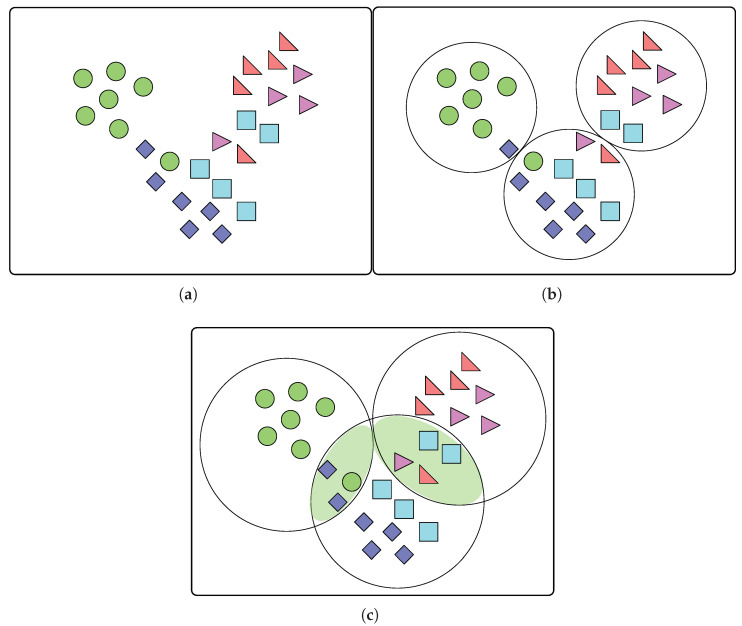
(**a**) All data *X* without clustering, (**b**) partitioning of *X* by *k*-means clustering, and (**c**) covering of *X* with ADS clustering. Each color represents the data with the same characteristics.

A *partition* of a set $X=\{x_{1},x_{2},\ldots ,x_{n}\}$ in mathematics implies a grouping of the elements in *X* into non-empty disjoint subsets $\left\{S_{1},S_{2},\ldots ,S_{k}\right\}$ such that every element in *X* is included in one and only one subset $S_{i}$ for some i∈{1,2,…,k}. The *k*-means method is an unsupervised clustering method that partitions data into *k* clusters to minimize the variance of each group and distance difference. The *k*-means method first selects *k* data randomly and sets them as the centroids of *k* clusters. Subsequently, the data are assigned to groups corresponding to the closest centroids. Once all data are grouped, the centroids of the clusters are recalculated. This procedure is repeated until the clusters of data no longer change. Specifically, the *k*-means method finds *k* clusters that minimize the following variance:

$$\min_{S_{i}}\sum_{i=1}^{k}\sum_{x_{j}\in S_{i}}\left||x_{j}-O_{i}\right|{|}^{2},$$

where $O_{i}$ is the centroid of the *i*-th cluster, $S_{i}$. Note that $\left\{S_{1},S_{2},\ldots .S_{k}\right\}$ is a partition of *X*; therefore, $S_{i}\cap S_{j}=\emptyset$ for i≠j and $\cup_{i=1}^{k}S_{i}=X$. For example, when data similar to that shown in Figure 2a are provided, the *k*-means method partitions them and introduces *k* clusters, as illustrated in Figure 2b.

Although the *k*-means method is efficient, it has certain limitations. First, the number of clusters, *k*, is a key hyperparameter and should be specified before model training. Section 4.2 introduces a preprocessing method to determine the optimal *k* from empirical data. Second, the error may converge to a local minimum instead of the global minimum, and the method is easily affected by outliers [17,18]. Third, and most importantly, because the *k*-means method *partitions* data into disjoint clusters, some data near the boundaries of the clusters may not be assigned to appropriate clusters. For instance, in Figure 2b, one blue rhombus in the left-most cluster and two sky blue squares in the right-most cluster match that in the middle better. In addition, the two pink and orange triangles in the cluster in the middle match the right-most cluster.

#### 3.1.2. Adaptive Data Selection

To address the limitations of the *k*-means method, we consider an ADS method. Given a dataset *X* (called the *universe*) and a family Ω of the subsets of *X*, ADS introduces a *cover* and a subfamily $\{C_{i},i=1,2,\ldots \}\subseteq \Omega$ of the subsets of *X*, whose union is the entire universe *X*. The cover for *X* in ADS is constructed as follows.

1. For an arbitrary but fixed point $x_{1}\in X$, $x_{1}$ is set as $O_{1}$, and the distance between $O_{1}$ and each point in *X* is measured. A subset $C_{1}\subset X$ is defined by the points in *X* within the *r*-neighborhood of $O_{1}$ for r>0. In this study, the Euclidean $L_{2}$ distance is used.
2. For an arbitrary but fixed point $x_{2}\in C_{1}^{c}=X\backslash C_{1}$, the complement of $C_{1}$, $x_{2}$, is set as $O_{2}$, and the distance between $O_{2}$ and each point in *X* is measured. A subset $C_{2}\subset X$ is defined by the points in *X* within the *r*-neighborhood of $O_{2}$. Because all points in *X* except $C_{1}^{c}$ are considered, $C_{1}$ and $C_{2}$ may not be disjoint.
3. For an arbitrary but fixed point $x_{3}\in {\left(C_{1}\cup C_{2}\right)}^{c}=X\backslash \left(C_{1}\cup C_{2}\right)$, the complement of $C_{1}\cup C_{2}$, $x_{3}$, is set as $O_{3}$, and the distance between $O_{3}$ and each point in *X* is measured. A subset $C_{3}\subset X$ is defined by the points in *X* within the *r*-neighborhood of $O_{3}$. Because all points in *X* are considered, $C_{1}\cup C_{2}$ and $C_{3}$ may not be disjoint.
4. Generally, when *i* sets $\left\{C_{1},C_{2},\ldots ,C_{i}\right\}$ are constructed, for an arbitrary but fixed point $x_{i+1}\in {\left(\cup_{k=1}^{i}C_{k}\right)}^{c}=X\backslash \cup_{k=1}^{i}C_{k}$, $x_{i+1}$ is set as $O_{i+1}$, and the distance between $O_{i+1}$ and each point in *X* is measured. A subset $C_{i+1}\subset X$ is defined by the points in *X* within the *r*-neighborhood of $O_{i+1}$. Note that $C_{i+1}$ may not be disjoint with $\cup_{k=1}^{i}C_{k}$.
5. This procedure is repeated until the union of the subsets includes *X*. If *n* subsets $C_{1},C_{2},\ldots ,C_{n}$ are constructed and the union of these *n* subsets is *X*, then the collection, $C=\{C_{1},C_{2},\ldots ,C_{n}\}$, is the cover of *X*.

Figure 2c shows the cover constructed from ADS for the data presented in Figure 2a. A preliminary version of ADS was proposed in [19].

### 3.2. Construction of a Group of ML Methods

A dataset *X* is split into training data $X_{tr}$ and test data $X_{ts}$. In this study, 80% of *X* is set as $X_{tr}$. A typical ML or DL method constructs one model that best describes the data in $X_{tr}$. The novelty of this study is the construction of a *group* of models for the data in $X_{tr}$, which is realized as follows. Suppose that the training data $X_{tr}$ is split into $C=\{C_{1},C_{2},\ldots ,C_{n}\}$ using the *k*-means or ADS method. Specifically, C is the partition of $X_{tr}$ if the *k*-means method is used, and it is a cover of $X_{tr}$ if the ADS method is used.

Subsequently, for each subset $C_{i}$, where i=1,2,…,n, a ML algorithm finds the parameters and hyperparameters using only the data in $C_{i}$ and constructs one model $M_{i}$ corresponding to $C_{i}$. Finally, *n* models $M=\{M_{1},M_{2},\ldots ,M_{n}\}$ are constructed from $C=\{C_{1},C_{2},\ldots ,C_{n}\}$. Note that the data in $C_{i}$ and $C_{j}$ for different i≠j may have different optimal parameters and hyperparameters, and this procedure allows different models $M_{i}$ and $M_{j}$ for different subsets, which is another novelty of this study.

When the training process is completed, the following procedure is performed on the test data.

For each data point *x* in the test dataset, $X_{ts}$,

1. The distance between *x* and the centroid, $O_{i}$, of each $C_{i}\in C$,
  (3) $$\begin{array}{c} d\left(x,O_{i}\right)=∥x-O_{i}∥ \end{array}$$
  is measured for i=1,2,…,n
2. The subset with the closest distance, e.g., $C_{i^{*}}$, is identified, which minimizes the distance as follows:
  (4) $$\begin{array}{c} d\left(x,O_{i^{*}}\right)=\min_{i}\left\{d\left(x,O_{i}\right)\;,\;i=1,2,\ldots ,n\right\} \end{array}$$
3. The ML model, $M_{i^{*}}$, trained with $C_{i^{*}}$ is used to perform regression to predict the output of *x*.

**Figure 3:**
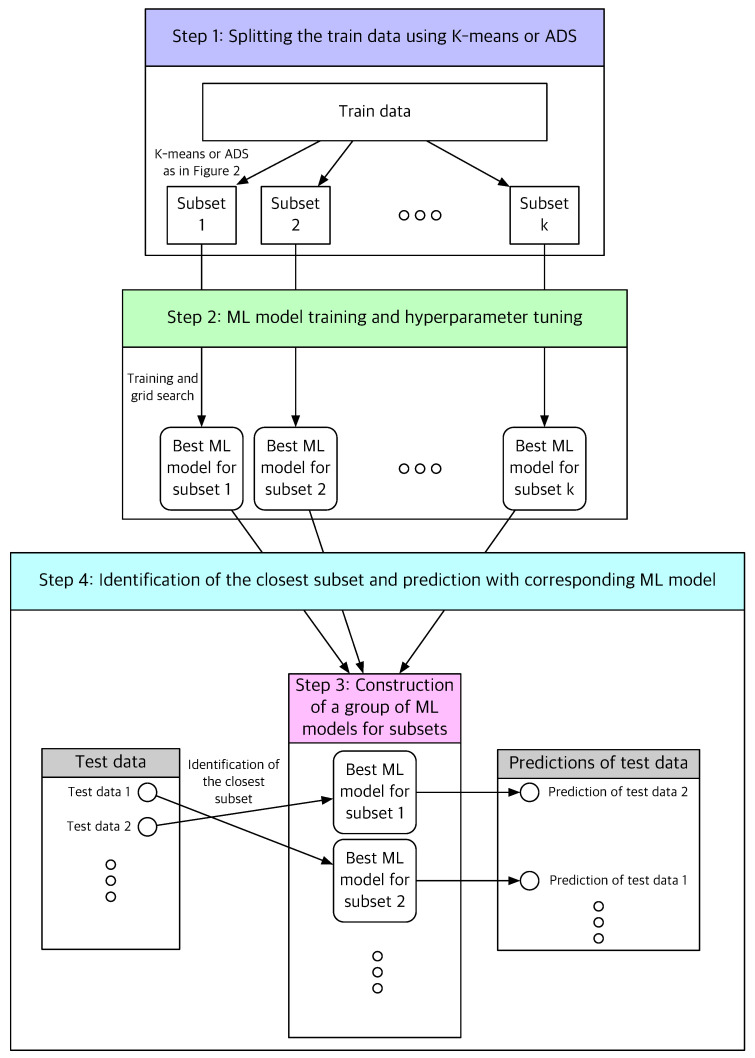
Schematic of the proposed ML-based prediction method using unsupervised clustering methods.

Figure 3 illustrates the procedure of the proposed method. In this paper, regression using *k*-means clustering is called *learning with k-means clustering*, whereas regression using ADS clustering is referred to as *learning with ADS clustering*. Regression without clustering, i.e., regression with all training data and a single training model, is called *learning without clustering*.

The algorithms of learning with *k*-means clustering and ADS clustering are summarized in Algorithms 1 and 2, respectively.
**Algorithm 1:***k*-means clustering-based learning algorithm
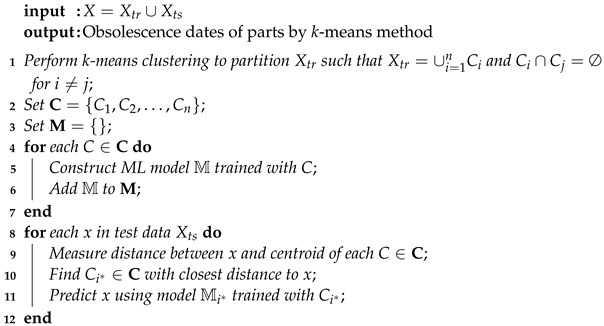


**Algorithm 2:** ADS clustering-based learning algorithm

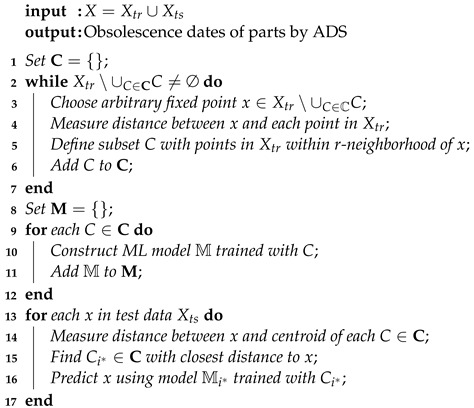



## 4. Data and Hyperparameters

In this study, a case study is performed based on an actual diodes dataset in Section 4.1 to test the performance of the proposed method in obsolescence forecasting. Section 4.2 shows the hyperparameters used in the experiments. Those for unsupervised learning are considered in Section 4.2.1 and those for supervised learning in Section 4.2.2.

### 4.1. Data Collection and Problem Description

Because the accurate prediction of component obsolescence reduces maintenance costs, many electronic component manufacturers and the defense industry have developed obsolescence forecasting systems. Several companies such as RAC, Z2Data, IHS, and QTEC provide their own obsolescence-related information; however, the detailed methodologies and algorithms are not disclosed. Additionally, information on the expected discontinuation dates has large error ranges. Thus, we propose the construction of a suitable prediction algorithm.

First, we build an actual dataset for this study as follows. The part numbers of the discontinued parts were obtained from QTEC, and their detailed characteristics and specifications were obtained from Z2Data. Among the available data, 2366 parts from the category of Zener diodes were selected and used as the dataset. Although parts from the other categories, such as varactors and bridge rectifier diodes, are available, the amount of information is insufficient for ML. Because the parts produced by different manufacturers have different specifications and characteristics in terms of the content and format, detailed technical information in the data sheets and test reports was thoroughly reviewed and standardized. The data of Zener diodes include 16 numeric and 21 categorical features. Table 2 lists their features and data types.

The *last time buy* (LTB) is the last order that is placed with a component supplier when the production of a component is to be terminated, and the obsolescence date in this study is the LTB date. Since the LTB date is an absolute date, the ML regression algorithms in this study use the *duration* between the part introduction date and the LTB date as the target variable. Once this relative date is predicted, the predicted obsolescence date is obtained by adding the predicted value to the part introduction date. The information about the part introduction dates is obtained from several services such as QTEC, IHS, or Z2Data. There are some parts whose introduction dates from those services are different, and the most recent date is selected as the introduction date in such cases.

Table 3 summarizes an example of Zener diode data. For simplicity, the values of only a few important features are presented.

Table 4 presents the descriptive statistics of the numeric features. *count*, *mean*, and *std* represent the number, mean, and standard deviation of the data, respectively. *min* and *max* are the minimum and maximum values, respectively; 25%, 50%, and 75% are quartiles, which divide the data points into four parts.

**Figure 4:**
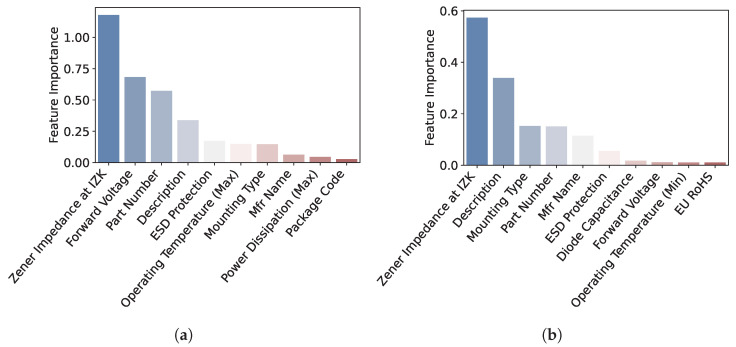

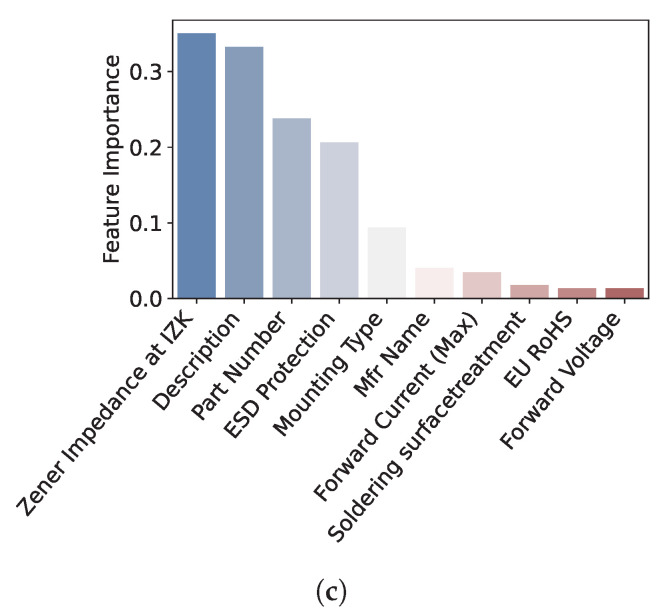
Feature importance from (**a**) DT, (**b**) RF, and (**c**) GB.

The features in Table 2 have their own significance when used in various ML methods. In feature importance, techniques assign scores to features based on their contributions to the prediction of the target variable. In this study, it is measured based on permutation feature importance, which represents the decrease in the model score when one feature value is randomly shuffled. This procedure breaks the relationship between the target and a feature, and thus, a decrease in the model score indicates the degree of model dependence on that feature. The features are standardized to zero mean and unit variance before the feature importance process, and the $R^{2}$ score is used as the scoring parameter of the importance function. Figure 4a shows the top ten importance features when the DT method is employed. The importance features are different for different methods, as shown in Figure 4b,c, which presents the top ten importance features when the RF and GB methods are used, respectively. Because the amount of data is small, dimensionality reduction based on the feature importance is not used in this study.

### 4.2. Hyperparameters

#### 4.2.1. Hyperparameters for Unsupervised Learning Algorithms

**Figure 5:**
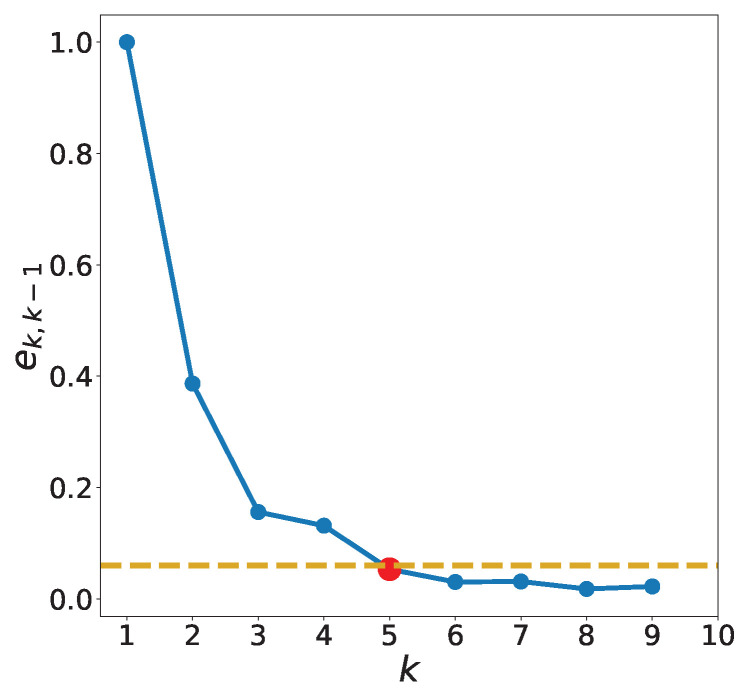
The blue solid line represents the accuracy, $e_{k,k-1}$ in (6), of the DT method for different *k* values of *k*-means for Zener diodes. The yellow dotted line represents the $e_{k,k-1}=0.06$, and the intersecting red dot represents the optimal value $k^{*}=5$.

Both the unsupervised clustering methods—*k*-means and ADS—have hyperparameters. For the *k*-means method, the appropriate number of clusters, *k*, needs to be estimated. It can be determined using the elbow method based on the sum of the squared errors or the silhouette method based on the silhouette score. Because the use of the *k*-means methods is aimed at helping in prediction, we select the optimal *k* based on the following preliminary regression stage. We use the DT method with the training data and observe the prediction accuracy as *k* increases. The DT method is employed in preprocessing because it is rapid, intuitive, and reasonably accurate. We determine the change in the error, $|e_{k}-e_{k-1}|$, where $e_{k}$ is the mean relative error (MRE) defined in Section 5 with *k* clusters, and select the *k* for which the change is sufficiently small. Specifically, the optimal $k=k^{*}$ is chosen as follows:

(5) $$\begin{array}{c} k^{*}=\arg\min_{k}\left\{e_{k,k-1}<h\right\}, \end{array}$$

where

(6) $$\begin{array}{c} e_{k,k-1}\equiv \alpha \left(e_{k}-e_{k-1}\right) \end{array}$$

and *h* is a predefined threshold. $\alpha ={\left(e_{2}-e_{1}\right)}^{-1}$ is introduced to avoid dataset dependency. The minimum value is considered in (5) because a small *k* reduces the computational cost. Figure 5 shows $e_{k,k-1}$ in (6) for several *k* values. In this study, h=0.06 is used, and the corresponding optimal *k* for the dataset is $k^{*}=5$.

In ADS, the radius, *r*, of a cluster is an important hyperparameter. Let *R* be the maximum distance between two arbitrary points in the training data, $X_{tr}$, i.e.,

$$R\equiv \max\{d\left(x_{i},x_{j}\right),\forall x_{i},x_{j}\in X_{tr}\}.$$

**Figure 6:**
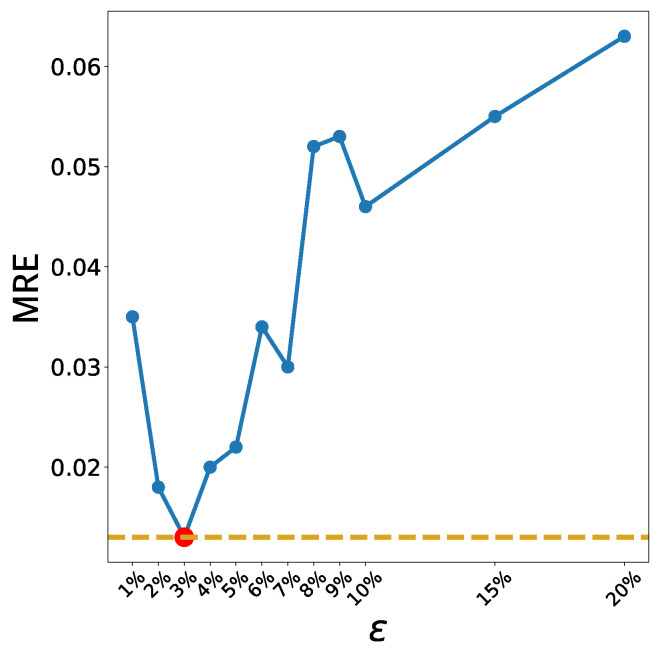
The blue solid line represents the accuracy (MRE) of DT for different radii of ADS for Zener diodes. The yellow dotted line represents the minimum value of MRE, and the intersecting red dot represents the optimal value $\epsilon^{*}=0.03$.

If r=R, then a single cluster includes all data in $X_{tr}$ regardless of the centroid. Therefore, we let r=ϵR for 0<ϵ<1 and calculate the MRE for various values of ϵ. The value minimizing the MRE is selected as the optimal value. Similar to the *k*-means method, DT is used to estimate the prediction accuracy, and the results are presented in Figure 6. The MRE is minimized when $r=\epsilon^{*}R$ with $\epsilon^{*}=0.03$, which is the value of ϵ used for the ADS method in this study. Table 5 lists the hyperparameters for the unsupervised clustering algorithms.

#### 4.2.2. Hyperparameters for Supervised Learning Algorithms

The hyperparameters of the supervised ML and DL methods used in this study are listed in Table 6. The left-most column contains the names of the parameters defined in the Scikit library [20]. The column in the middle provides the definition of each hyperparameter, and the right-most column lists the values of the hyperparameters considered in this study. Four hyperparameters are considered for the DT and GB methods and three for the RF, DNN, and RNN methods. For instance, max_depth for the DT method represents the maximum depth of the tree and has one of five values: None, 2, 4, 6, and 8. If None, then the nodes are expanded until all leaves are pure or until all leaves contain fewer than min_samples_split samples. A grid of all the possible hyperparameter combinations is created, and the best hyperparameters are obtained. Model tuning with grid search is performed for all models using the hyperparameter values in Table 6.

**Figure 7:**
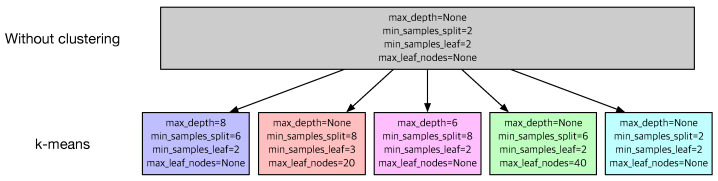
The values of the hyperparameters of DT without clustering and with *k*-means.

Figure 7 compares the optimal hyperparameters of DT without clustering and 5 sets of optimal hyperparameters for 5 subsets from *k*-means. Each subset from *k*-means has different values of hyperparameters. For instance, *max_depth* for the leftmost data is 8 while that for the data in the middle is 6. Different parameters and hyperparameters for each subset confirm that a single DT model may not be appropriate for the given dataset and that a group of DT models from the proposed method will produce better predictions.

## 5. Results and Discussion

We show the performance of the proposed method for the obsolescence prediction of Zener diodes. Section 5.1 defines the metrics used for the measurements of prediction errors. Section 5.2 validates the reliability of the proposed method by considering the prediction of obsolescence dates of non-discontinued parts. The errors of the proposed method are compared with those of naive statistics in Section 5.3 and with those of other ML methods in Section 5.4. Confidence intervals of the errors are considered in Section 5.5.

### 5.1. Metrics

In this study, two distance metrics are considered. The first is the MRE defined in (7).

(7) $$\begin{array}{c} \mathrm{MRE}=\frac{1}{n}\sum_{i=1}^{n}\left|\frac{y_{i}-{\tilde{y}}_{i}}{y_{i}}\right|, \end{array}$$

where $y_{i}$ is the actual target value, and ${\tilde{y}}_{i}$ is its predicted value; and *n* is the number of points used to make the predictions. The second metric is the root mean squared relative error (RMSRE) defined in (8).

(8) $$\begin{array}{c} \mathrm{RMSRE}=\sqrt{\frac{1}{n}\sum_{i=1}^{n}{\left(\frac{y_{i}-{\tilde{y}}_{i}}{y_{i}}\right)}^{2}} \end{array}$$

The MRE and RMSRE are similar to the mean absolute error (MAE) and the root mean squared error (RMSE), respectively; however, the errors from the latter two might be misleading because they depend on the scale of the values. By contrast, the former metrics measure relative errors without depending on the value ranges and, thus, are used in this study.

Instead of ML methods, standard statistical models may be used to perform regression analysis of the obsolescence date. The sample mean of the observed obsolescence dates in the training data is defined as

(9) $$\begin{array}{c} S_{\mathrm{TATISTIC}}=\frac{1}{N_{tr}}\sum_{i=1}^{N_{tr}}y_{i}, \end{array}$$

where $N_{tr}=\left|X_{tr}\right|$ is the cardinality of the training data. This is one of the possible statistics and used as a benchmark in this study.

### 5.2. Reliability of the Proposed Method

**Figure 8:**
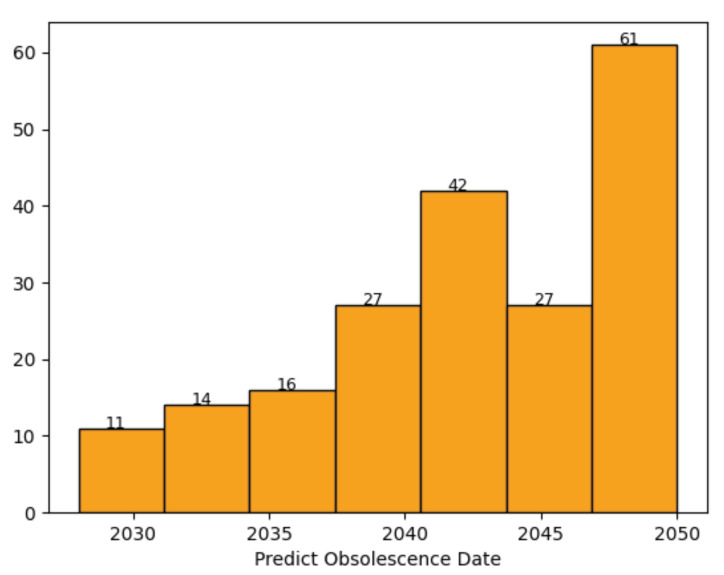
The histogram of predicted obsolescence dates for non-discontinued data using the proposed method with ADS and DT.

In order to check whether the proposed method is reliable, the method has been applied to the obsolescence prediction of *non-discontinued* Zener diodes as well. We do not know when the correct obsolescence date will be for the non-discontinued parts, but if the obsolescence dates are predicted to be in the past, the proposed method will be questionable. We collected 198 non-discontinued Zener diodes, and Figure 8 shows the histogram of their predicted obsolescence dates when the proposed method is applied with ADS and DT. All the obsolescence prediction dates are in the future, and none are in the past. Similar results are obtained when the proposed method is applied with other methods, which confirms that the proposed scheme is reliable and not questionable.

### 5.3. Comparison with Naive Statistics

**Figure 9:**
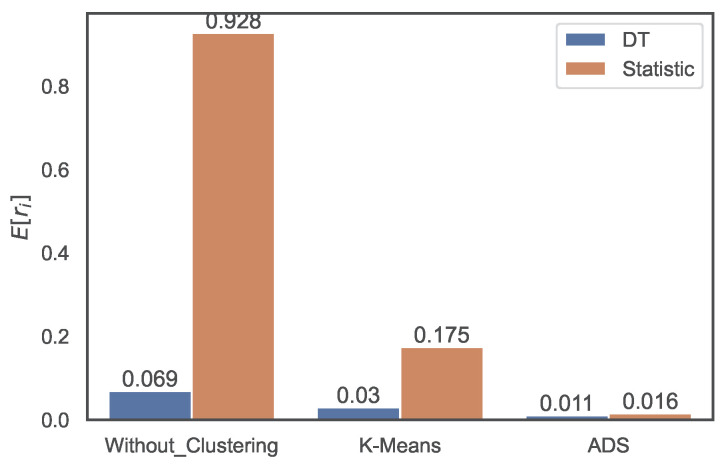
Distributions of MRE values of predictions using the DT method and naive statistic without clustering, with *k*-means clustering, and with ADS clustering.

First, we compare the prediction accuracy of the ML methods with that of naive statistics. Figure 9 shows the average $E[r_{i}]$ of the relative errors of the prediction, which is defined as

(10) $$\begin{array}{c} r_{i}\equiv \frac{y_{i}-{\tilde{y}}_{i}}{y_{i}} \end{array}$$

when the DT method and the naive statistic are used. The prediction error of the DT method without clustering is 0.069, and the error of the naive statistic is 0.928, which is 13 times larger than the former. The error of the DT method with *k*-means clustering is 0.030, and that of the naive statistic is five times larger. Similar improvements are achieved using ADS clustering. As is evident from the figure, clustering improves the regression. The prediction error of the DT method without clustering is 0.069, which decreases to 0.030 with the *k*-means clustering and further reduces to 0.011 with the ADS clustering. Moreover, the error of the naive statistic is improved from 0.928 without clustering to 0.175 with the *k*-means clustering and 0.016 with the ADS clustering.

**Figure 10:**
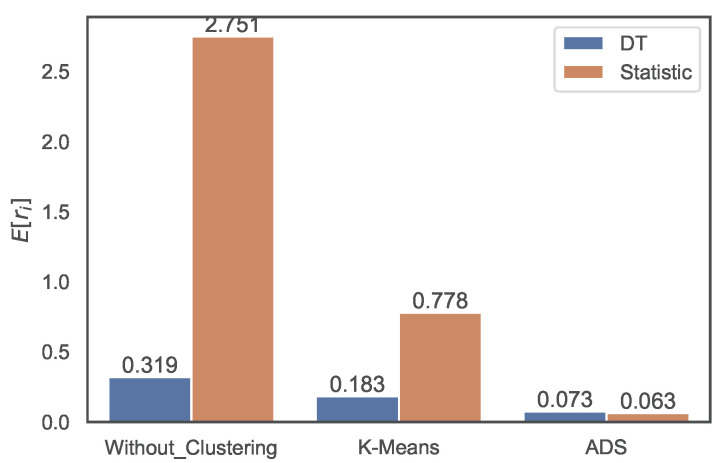
Distributions of RMSRE values of predictions using DT method and naive statistic without clustering, with *k*-means clustering, and ADS clustering.

Figure 10 shows the distribution accuracy in terms of the RMSRE, and the results are similar to those of the MRE in Figure 9. The RMSRE error of the DT method without clustering is 0.319 and the error of the statistic is 2.751, which is eight times larger than the former. The error of the DT method with the *k*-means clustering method is four times smaller than that of the naive statistic. Additionally, the error of the DT method without clustering decreases to 0.183 with *k*-means clustering and further reduces to 0.073 with ADS clustering. The naive statistic is also improved by the clustering-based regression.

**Figure 11:**
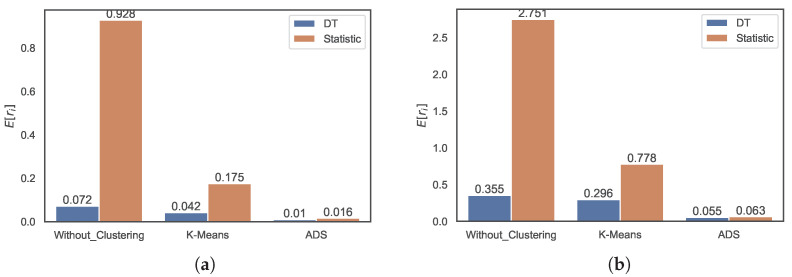
Distributions of (**a**) MRE and (**b**) RMSRE values of predictions using the RF method and naive statistic without clustering, with *k*-means clustering, and with ADS clustering.

Figure 11 shows the distributions of the MRE and RMSRE of the RF method. Similar to the corresponding distributions of the DT method presented in Figure 9 and Figure 10, the prediction accuracy using the ML RF method is better than that of the naive statistic, and the prediction accuracy with clustering is better than that without clustering. For instance, the MRE of the statistic without clustering is approximately 13, 4, and 16 times larger than those of the RF method, RF method with the *k*-means clustering, and RF method with ADS clustering, respectively. Additionally, the MRE of the RF method without clustering is 0.072, which is reduced to 0.042 with the *k*-means clustering and further decreased to 0.010 with the ADS clustering. Similar trends are observed for the RMSRE. The results from other ML methods, which are not presented herein, showed similar trends and are omitted. These results indicate that ML methods yield considerably better results than the naive statistic, and clustering improves the regression.

### 5.4. Comparison of ML Methods

This subsection discusses the effects of clustering. The prediction accuracies of various ML methods with and without clustering are compared to determine the method yielding the optimal regression result. Because ML methods are superior to the naive statistic, as discussed in the previous section, the results from the statistic are omitted, and only those obtained using the ML methods are presented.

**Figure 12:**
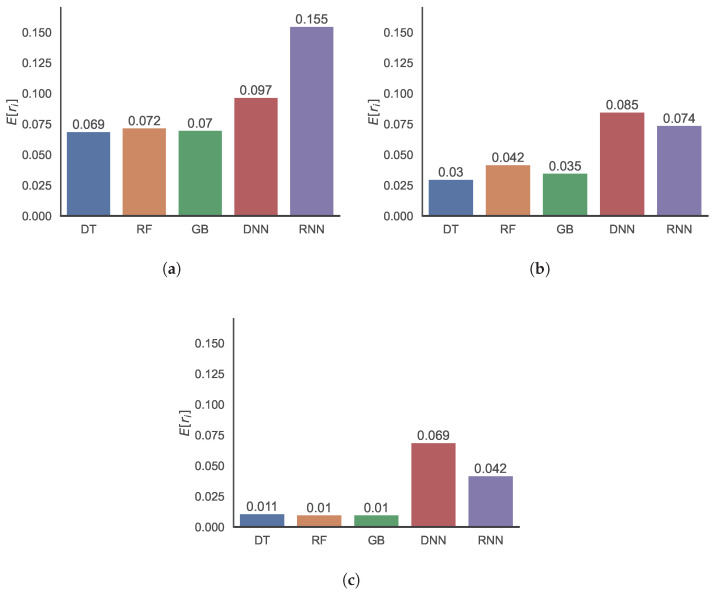
Distributions of MRE values of predictions using DT, RF, GB, DNN, and RNN methods (**a**) without clustering, (**b**) with *k*-means clustering, and (**c**) with ADS clustering.

Figure 12 shows the prediction errors in terms of the MREs of various ML methods, namely DT, RF, GB, DNN, and RNN. Figure 12a–c shows the distributions when the methods are used without clustering, with *k*-means clustering, and with ADS clustering, respectively. The three shallow learning methods—DT, RF, and GB—yield good prediction accuracy, and the results of the two DL methods—DNN and RNN—are slightly inferior. DL typically results in better results than conventional ML methods; however, the insufficient amount of data in this study results in inferior performance.

**Figure 13:**
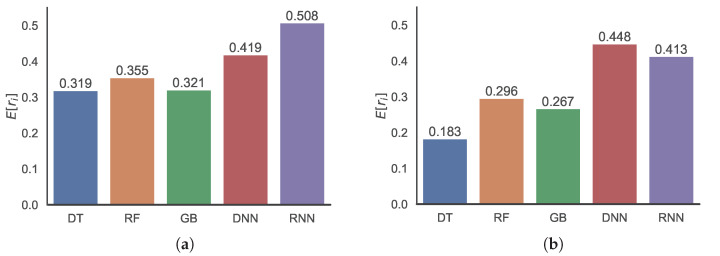

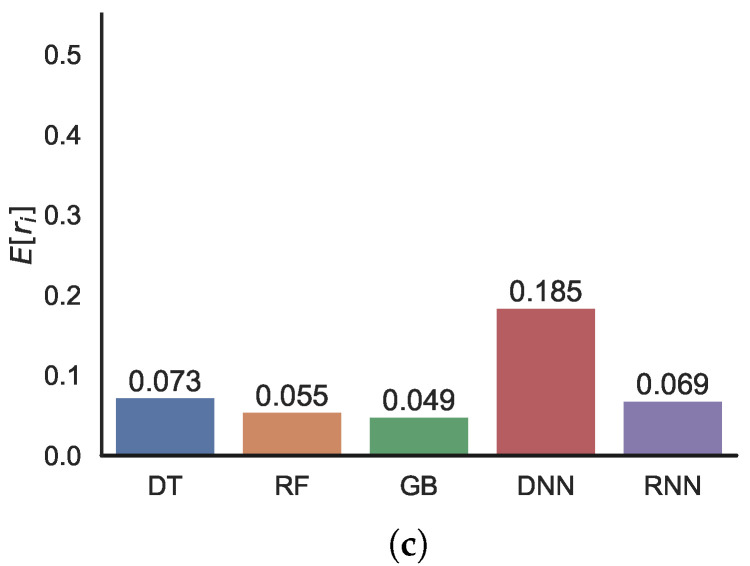
Distributions of RMSRE values of predictions using DT, RF, GB, DNN, and RNN methods (**a**) without clustering, (**b**) with *k*-means clustering, and (**c**) with ADS clustering.

Figure 13 shows the prediction errors in terms of the RMSRE, and the trends are similar to those of the MRE, as presented in Figure 12. Notably, the RMSREs are relatively larger than the MREs owing to insufficient data, and the RMSRE is more dependent on such values than the MRE. Both Figure 12 and Figure 13 show that clustering improves the prediction accuracy of the ML methods. Moreover, the ADS-based clustering, the results of which are presented in Figure 12c and Figure 13c, outperforms the *k*-means clustering, the results of which are shown in Figure 12b and Figure 13b.

### 5.5. Confidence Intervals

**Figure 14:**
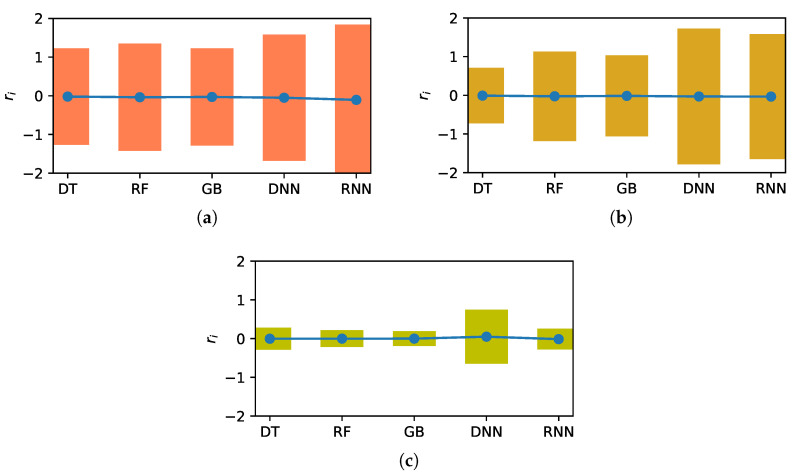
Comparison of 95% confidence intervals of predicted values using various methods. (**a**) Without clustering, (**b**) with *k*-means clustering, and (**c**) with ADS clustering.

Figure 14 presents 95% confidence intervals of the errors of the various methods. The intervals for the ML methods without clustering are larger than those for those with clustering, and the ADS-based clustering reduces the widths more than the *k*-means clustering.

Table 7 lists the widths of the 95% confidence intervals shown in Figure 14. Evidently, methods with clustering have a smaller confidence interval width than that without clustering, and the confidence interval width with ADS is much smaller than that with *k*-means. Therefore, clustering yields estimates that are more stable and accurate, and the estimates with the ADS clustering are superior to those with the *k*-means clustering.

## 6. Conclusions

Given a labeled dataset, a supervised ML algorithm finds parameters and hyperparameters from the data and builds a single model for classification or regression. When the dataset is highly nonlinear, however, a single model may not be sufficient for accurate modeling or prediction. In this study, we propose a method to split the dataset into its subsets and identify the parameters and hyperparameters for each subset. We apply two unsupervised clustering methods to split the dataset to its subsets. One is *k*-means clustering, whose subsets are mutually disjoint and partition the whole dataset. The other is ADS, whose subsets have non-empty intersections. Subsequently, supervised learning builds one model for each subset and eventually forms a group of ML models for the whole dataset. For each data point in the test dataset, one identifies a subset that best describes the characteristics or pattern of the data point and applies the ML model corresponding to the subset for the prediction.

Supervised learning is an ML approach for the classification or regression of the labeled data, while unsupervised learning is for clustering, dimensionality reduction, or association of the unlabelled data. It is the novelty of the study to introduce a method to combine unsupervised clustering with supervised regression to develop a prediction model with improved accuracy and efficiency.

Although both *k*-means and ADS improved the prediction accuracy, the ADS-based regression outperformed the *k*-means-based method. This is because the covers from the ADS allow non-empty intersections, enabling better handling of the data near the boundaries of the subsets than with the *k*-means method. The results of this study are novel because they demonstrate that unsupervised clustering methods can improve supervised learning algorithms.

For future research, we can consider several directions. First, the proposed method showed poor results in this study when combined with DNN and RNN methods because the electrical component data considered in this study were insufficient for DL methods. If sufficient data are obtained, the proposed method combined with DL methods is expected to yield improved predictions in terms of accuracy and efficiency.

Combining unsupervised clustering with supervised learning to improve the prediction accuracy can be extended and validated in various fields. Great effects are expected when the dataset is highly nonlinear, as in financial datasets. Thus, the application of the proposed method to financial optimization or derivatives pricing will be an interesting problem. We hope that our study provides a good reference for the predictions not only in the area of DMSMS but also in many other similar areas.

ADS is an unsupervised learning method that was developed very recently. More detailed analysis of its properties and usefulness in clustering or dimensionality reduction should be studied.

## Figures and Tables

**Table 2:** Features of data of Zener diodes.

Type	Features
Numeric	Power Dissipation, Reverse Zener Voltage (Min), Reverse Zener Voltage (Max), Test Current, Zener Impedance (Max), Zener Impedance at IZK, Maximum Zener Current, Reverse Leakage Current at VR, Reverse Voltage, Forward Voltage, Voltage Tolerance, Forward Current, Diode Capacitance, Operating Temperature (Min), Operating Temperature (Max), Number of Terminals
Categorical	Part Number, Mfr Name, Description, Polarity, ESD Protection, Temperature Coefficient, EU RoHS, Halogen Free, Package Code, Soldering Surface Treatment, Mounting Type, JESD-30 Code, Package Body Material, Package Shape, Package Style, Terminal Form, Terminal Position, Temperature Grade, Part Status, Part Introduction, and Obsolete Date (LTB Date)

**Table 3:** Example of Zener diode data.

Feature	Value
Zener Impedance at IZK (Ω)	2000.0
Forward Voltage (V)	1.2
Part Number	1N4761A
Description	ZENER DIODE, 1 W, 75 V@3 MA, 5%
ESD Protection	Unknown
Operating Temperature (Max) (${}^{\circ }C$)	200
Mounting Type	Through Hole
Mfr Name	CENTRAL SEMICONDUCTOR CORP.
Power Dissipation (Max) (W)	1.0
Package Code	DO-41

**Table 4:** Statistics of certain numeric features of discontinued Zener diodes.

	Count	Mean	Std	Min	25%	50%	75%	Max
Power Dissipation (Max) (W)	2366	2.52	2.49	0.12	0.50	1.0	5.00	10.0
Reverse Zener Voltage (Min) (V)	1051	26.70	36.66	1.80	5.60	12.4	28.50	190.0
Reverse Zener Voltage (Max) (V)	2366	42.23	52.44	1.80	8.53	20.0	53.16	270.0
Test Current (mA)	2366	32.61	61.26	0.25	5.00	10.0	30.00	640.0
Zener Impedance (Max) (Ω)	2366	110.81	240.05	1.00	9.00	30.0	100.00	2500.0
Zener Impedance at IZK (Max) (Ω)	1936	1129.16	1361.70	60.00	400.00	700.0	1300.00	8000.0
Maximum Zener Current (mA)	1244	200.44	288.66	1.54	31.60	85.0	264.00	2380.0
Reverse Leakage Current @ VR (uA)	2366	8.73	22.78	0.05	1.00	2.0	5.00	300.0
Reverse Voltage (V)	2366	31.16	39.76	0.50	6.00	15.0	38.80	206.0
Forward Voltage (V)	1811	1.29	0.21	0.90	1.20	1.2	1.50	1.5
Voltage Tolerance (Max) (%)	2366	4.62	2.56	1.00	2.38	5.0	5.00	20.0
Forward Current (Max) (mA)	1811	437.88	395.93	2.00	200.00	200.0	1000.00	1000.0
Diode Capacitance @ f = 1 MHz, VR = 0 V (pF)	157	178.23	148.24	19.00	70.00	130.0	225.00	450.0
Operating Temperature (Min) (${}^{\circ }C$)	2366	−62.52	4.32	−65.00	−65.00	−65.0	−65.00	−55.0
Operating Temperature (Max) (${}^{\circ }C$)	2366	171.56	16.36	125.00	150.00	175.0	175.00	200.0
Number of Terminals	2366	1.98	0.49	0.00	2.00	2.0	2.00	4.0

**Table 5:** Hyperparameters for unsupervised learning algorithms.

Method	Hyperparameter	Value
*k*-means	Number of clusters (*k*)	5
ADS	Radius of cover (*r*)	0.03R

**Table 6:** Hyperparameters for supervised learning algorithms.

DT	Definition	Values
max_depth	Maximum depth of tree	None, 2, 4, 6, 8
min_samples_split	Minimum number of samples required to split internal node	2, 4, 6, 8
min_samples_leaf	Minimum number of samples required to be at leaf node	2, 3, 4, 5
max_leaf_nodes	Maximum number of terminal nodes	20, 40, 60, unlimited
RF	Definition	Values
min_samples_split	Minimum number of samples required to split internal node	2, 3, 4, 5
n_estimators	Number of trees in forest	100, 150, 200
max_features	Number of features to consider when determining best split	auto, sqrt, log2
GB	Definition	Values
learning_rate	Learning rate reduces contribution of each tree	0.01, 0.1, 0.2
subsample	Fraction of samples to be used for fitting individual base learners	0.5, 0.6, 0.7, 0.8, 0.9, 1
n_estimators	Number of boosting stages to perform	100, 200, 300, 400, 500
max_depth	Maximum depth of individual regression estimators	2, 4, 6, 8, 10
DNN	Definition	Values
unit	Processors between input and output units in connectionist system	32, 64
optimizer	Adjusts model weights to maximize loss function	Adam, Nadam, RMSprop
dropout	Technique by which randomly selected neurons are ignored during training	0, 0.1, 0.01
RNN	Definition	Values
unit	Processors between input and output units in connectionist system	32, 64
optimizer	Adjusts model weights to maximize loss function	Adam, Nadam, RMSprop
dropout	Technique by which randomly selected neurons are ignored during training	0, 0.1, 0.01

**Table 7:** Comparison of widths of 95% confidence intervals of predicted values using various methods.

	Without_Clustering	*k*-Means	ADS
DT	1.249144	0.716651	0.285660
RF	1.384982	1.157487	0.216856
GB	1.253258	1.046753	0.190099
DNN	1.630955	1.753372	0.699891
RNN	1.946344	1.614736	0.266980

## Data Availability

Data sharing not applicable.

## Abbreviations

The following abbreviations are used in this manuscript:

DT	decision tree
RF	random forest
GB	gradient boosting
DNN	deep neural network
RNN	recurrent neural network
MRE	mean relative error
RMSRE	root mean squared relative error
